# Time-based prospective memory functioning in mild cognitive impairment associated with Parkinson’s disease: relationship with autonomous management of daily living commitments

**DOI:** 10.3389/fnhum.2015.00333

**Published:** 2015-06-10

**Authors:** Alberto Costa, Silvia Zabberoni, Antonella Peppe, Francesca Serafini, Francesco Scalici, Carlo Caltagirone, Giovanni Augusto Carlesimo

**Affiliations:** ^1^Department of Clinical and Behavioral Neurology, IRCCS Santa Lucia FoundationRome, Italy; ^2^Università Niccolò CusanoRome, Italy; ^3^Medicina dei Sistemi, Università Tor VergataRome, Italy

**Keywords:** prospective memory, Parkinson’s disease, medication management, cognitive functions, daily living

## Abstract

**Objective**: Prospective memory (PM), that is, the ability to keep in memory and carry out intentions in the future, is reported to be impaired in individuals with Parkinson’s disease (PD). PM failure may be also associated with reduced daily living functioning in these patients. Little is known, however, about the relationship between mild cognitive impairment (MCI) and time-based PM functioning in PD patients and the possible impact of PM deficits on patients’ autonomy in daily living. Here we aimed to investigate whether MCI associated with PD affects time-based PM. We also wished to determine whether PM impairment accounts for reduced autonomous management of medication in these patients.

**Method**: The study included 48 PD patients with MCI, 33 PD patients without cognitive disorders (PDN) and 20 healthy controls. The time-based PM procedure required that subjects perform an action after a fixed time. The PM procedure was incorporated in the standard neuropsychological assessment. One score was computed for the ability to retrieve the intention (prospective component) and one for remembering the action to be executed (retrospective component). The Pill Questionnaire was administered to assess the ability to manage medication.

**Results**: PD patients with MCI performed less accurately in the PM procedure than HC and tended to perform poorer than PDN. Moreover, in PD patients with MCI, accuracy on the prospective component of the PM task and performance on the Modified Card Sorting Test significantly predicted the ability to manage medication.

**Conclusions**: Results document that reduced efficiency of time-based PM processes in PD is specifically related to the presence of MCI. The same data indicate that PM weakness may be associated with impaired daily living functioning and decreased autonomy.

## Introduction

Prospective memory (PM), that is, the ability to keep in mind and carry out future intentions, is consistently impaired in individuals with Parkinson’s disease (PD). In fact, studies using both event-based paradigms, which require subjects to execute a delayed action at the occurrence of an environmental cue, and time-based tasks, which require subjects to execute a delayed action at the expiration of a certain time, document that PD patients perform significantly worse than healthy controls (Kliegel et al., 2011; Costa et al., 2013a; Foster et al., 2013). There is also evidence that in this population PM impairment is related to dysexecutive symptoms (Kliegel et al., 2011; Costa et al., 2014) and a role has been suggested for the dysfunctioning of dopamine systems (Costa et al., 2008a).

Findings of a recent study also raised the question of whether PM weakness is tout-court related to PD in PD patients or whether it is the expression of cognitive impairment (Costa et al., 2015a). In particular, in that study PD patients with and without mild cognitive impairment (MCI) and healthy controls were administered an event-based task in focal (the PM target was processed in the ongoing task) and non-focal (the PM target was not processed during the ongoing task) conditions. Results documented that PD patients with MCI performed worse than both healthy controls and PD patients without MCI, who in turn performed comparably (Costa et al., 2015a). These findings have clinical relevance because they suggest that PM disorders may be a specific sign of MCI associated with PD. Indeed, as outlined by several authors MCI can be considered as a prodromal condition of different neurological syndromes with dementia (Petersen et al., 2001; Dubois et al., 2007a, 2010; Cummings et al., 2013). An elevated risk of dementia has also been reported for individuals with MCI associated with PD compared to individuals with PD without MCI (Litvan et al., 2012; Halliday et al., 2014). Therefore, clarifying whether PM impairments depend on MCI in PD is an important issue that could provide useful information for diagnosis and treatment.

Another open question concerns the possible relationship between PD patients’ PM weakness and reduced ability to self-manage daily living commitments. Indeed, PM impairment is reported to significantly affect functional activities in persons with various neurological diseases (Kinsella et al., 1996; Burgess et al., 2000; Schmitter-Edgecombe et al., 2009; Fish et al., 2010; Kliegel et al., 2011). In PD patients, Pirogovsky et al. (2012) found that decreased PM efficiency, as assessed by both self-report and performance-based tools, was significantly associated with reduced ability to successfully manage their own medication. In a recent investigation this relationship was also described in PD patients with MCI. In particular, in a sample of PD patients with single-domain and multiple-domain MCI event-based PM scores were found to significantly predict scores on the Pill questionnaire, a tool aimed at ascertaining whether PD patients are able to autonomously manage pharmacological therapy (Costa et al., 2015a). This was the first study directly aimed at exploring this issue, which is undoubtedly worthy of further investigation. Moreover, the possible effect of other factors such as depression, apathy and severity of extrapiramidal symptoms, which might significantly affect daily living management, was not taken into account.

To the best of our knowledge, time-based PM functioning has never been compared in PD patients with and without MCI. Moreover, its relationship with self-management of daily living commitments has never been investigated in individuals with MCI associated with PD. Therefore, this study was specifically aimed at investigating these issues. For this purpose, we administered a time-based PM procedure that was incorporated in the standard neuropsychological examination to two groups of PD patients with single- and multiple-domain MCI, a group of PD patients without MCI and a group of healthy controls. Based on previous data concerning event-based PM functioning, we predicted that PD patients with MCI, but not PD patients without MCI, would obtain lower PM scores than healthy controls.

To comply with the second aim of study, that is, to investigate the relationship between PD patients’ PM performance and their autonomy in daily living, a regression model was applied to the data. In particular, we evaluated the relative contribution of PM scores to the score variability on the Pill questionnaire (Dubois et al., 2007b), taking into account the weight of other relevant cognitive (i.e., global cognitive functioning, episodic memory and executive abilities), affective (i.e., depression and apathy) and motor (severity of extrapiramidal symptoms) variables. According to previous evidence of an association between PM failure and reduced daily living functioning also in PD patients (Pirogovsky et al., 2012; Costa et al., 2015a), we expected that PM performance would predict the ability of PD patients with MCI to autonomously manage their own medication.

## Materials and Methods

### Participants

We recruited 81 right-handed individuals with idiopathic PD and 20 right-handed healthy controls (HC; *F* = 17; *M* = 3); they participated in the study after giving their written informed consent. The study was approved by the Ethics Committee of Fondazione Santa Lucia.

Idiopathic PD was defined according to the United Kingdom PD Society brain bank criteria (Hughes et al., 1992). Diagnosis of MCI was made according to Litvan et al. (2012) criteria, specifically: (i) cognitive complaints corroborated by an assistant; (ii) performance score at least 1.5 SD below the normative population on two tests of a standardized neuropsychological screening battery (see below for a detailed description of the tests used). Exclusion criteria were: (i) the presence of major psychiatric disorders, neurological conditions other than PD, vascular brain lesions and major systemic or metabolic diseases that could affect cognitive status as assessed by neuropsychiatric, neuroradiological (CT or MR) and laboratory examinations; (ii) taking medications (other than dopaminergic drugs) that have an effect on brain functioning. According to Litvan et al. (2012), PD patients were classified as having MCI (single-domain MCI: *N* = 20 (*F* = 11; *M* = 9): 9 with amnestic and 11 with dysexecutive profiles; multiple-domain MCI: *N* = 28 (*F* = 10; *M* = 18): 19 with memory and executive impairments, 7 with executive and visual-spatial impairments, 1 with memory and visual-spatial impairments; 1 with memory, executive and visual-spatial impairments), and patients without cognitive disorders (PDN; *N* = 33; *F* = 15; *M* = 18). All PD patients were treated with levodopa, dopamine agonists (pramipexole, ropinirole, rotigotine) or monoamine oxidase inhibitor (rasagiline and selegiline; in Table 1 average dopamine equivalent dosages are reported for the three PD groups). The Unified PD Rating scale Part-III (Fahn and Elton, 1987) was administered to assess severity of extrapiramidal symptoms. The Activities of Daily Living (ADL) and Instrumental Activities of Daily Living (IADL; Lawton and Brody, 1969) and the Pill questionnaire (see below for a detailed description of this tool; Dubois et al., 2007b) were administered to assess patients’ ability to manage routine activities.

**Table 1:** **Social-demographic and clinical characteristics of the four experimental groups**.

	Healthy subjects *N* = 20	PD patients without MCI *N* = 33	PD patients with MCI *N* = 48	*F* values	*p* values
Age	66.0 (7.0)	63.4 (8.7)	66.0 (9.0)	1.02	0.37
Years of formal education	12.3 (3.6)	12.6 (3.0)	11.4 (4.3)	1.08	0.34
MMSE	29.4 (0.8)*	29.4 (0.8)**	28.5 (1.4)	8.13	0.001
BDI	6.5 (5.7)	8.7 (6.9)	8.7 (7.3)	0.65	0.52
AES	27.7 (4.6)	29.1 (8.9)	32.1 (6.9)	1.94	0.065
Pill questionnaire	—	3.79 (0.54)	3.08 (1.05)	6.90	0.001
IADL	—	7.4 (1.1)	6.8 (1.4)	1.76	0.074
Disease duration	—	6.9 (4.7)	6.3 (4.1)	0.41	0.52
UPDRS—Part III (“on” condition)	—	19.7 (13.5)	22.4 (11.7)	0.92	0.34
Levodopa equivalents	—	607 (230)	616 (259)	0.03	0.87

*Indicates a significant difference between healthy subjects and PD individuals with MCI, with a p value ≤0.01 as results from LSD post hoc tests; ** Indicates a significant difference between PD patients without MCI and the PD individuals with MCI with p < 0.01. *SDMCI, single domain mild cognitive impairment; MDMCI, multiple domains mild cognitive impairment; BDI, Beck Depression Inventory; AES, Apathy Evaluation Scale; IADL, Instrumental Activities of Daily Living; UPDRS, Unified Parkinson’s Disease Rating Scale*.

Inclusion criteria for HC included: (i) absence of subjective cognitive disturbances; (ii) MMSE score ≥26 (Measso et al., 1991). Exclusion criteria were: (i) performance 1.5 SD below the normative population on any test of the standardized neuropsychological screening battery; (ii) presence of current or previous neurological or psychiatric disorders, major systemic or metabolic diseases able to induce significant changes in cognition; and (iii) taking medications with an effect on brain functioning.

Table 1 presents the socio-demographic and clinical characteristics of the samples.

### Neuropsychological Test Battery

Standardized tests were administered to PD patients to assess episodic memory [Immediate and Delayed Recall of a 15-Word List (Carlesimo et al., 1996); Prose Recall (Carlesimo et al., 2002); Immediate and delayed reproduction of Rey’s Figure (Carlesimo et al., 2002)], attention and short-term memory [Digit Span and Corsi Block Tapping test Forward and Backward (Monaco et al., 2013); the Trail Making Test—Part A (Giovagnoli et al., 1996)], executive functions [Phonological Word Fluency (Carlesimo et al., 1996); Modified Card Sorting test (MCST; Nocentini et al., 2002); Raven’s Colored Progressive Matrices (Carlesimo et al., 1996); the Trail Making Test—Part B (Giovagnoli et al., 1996)], language [Objects and Verbs Naming subtests from the Neuropsychological Examination of Aphasia (Capasso and Miceli, 2001)], visual-spatial functions [Copy of Drawings and Copy of Drawings with Landmarks (Carlesimo et al., 1996); Copy of the Rey’s Figure (Carlesimo et al., 2002)].

### Evaluation of PD Patients’ Ability to Self-Manage Pharmacological Therapy

The Pill questionnaire (Dubois et al., 2007b) is an instrument administered to patients and their caregivers that investigates patients’ ability to manage their dopamine treatment. According to the task force of the Movement Disorder Society, this questionnaire assesses the impact of a decline in cognitive functioning on daily living, taking into account the possible effects of motor disorders. According to Dubois et al. (2007b) no impact of cognitive disorders on daily living is present if a patient is able to describe the drugs, doses and timing of therapy or in cases in which the patient needs some help from the examiner but the caregiver certifies that the patient can safely and reliably take his/her pills without supervision (score = 4 and 3, respectively). There is an impact on daily living if the caregiver reports that the patient cannot take the pills without supervision or if the patient is unable to describe (even with the help of the examiner) the drugs, doses and timing of dopamine therapy (scores of 2 and 1, respectively).

### Assessment of Severity of (Depression) and Apathy

The Beck Depression Inventory (Beck et al., 1961; Visser et al., 2006) and the Apathy Evaluation Scale—Patient Version (Marin et al., 1991; Leentjens et al., 2008) were also administered to assess severity of depression and apathy, respectively.

The neuropsychological assessment and the administration of self-report questionnaires was carried out on two different days over a one-week period.

### PM Task

#### Material

The material consisted of five different actions the subjects had to perform at the expiration of the established time (i.e., open the door, remember to turn on the computer, write their first and last name on a sheet of paper, give a pen back to the examiner, setting right the phone receiver). The examiner and the experimental subject were seated on opposite sides of a table on which the objects the subject had to use were located. A wall clock was placed to the right of the subject so that he had to turn his head to check it.

#### Procedure

**Figure 1:**
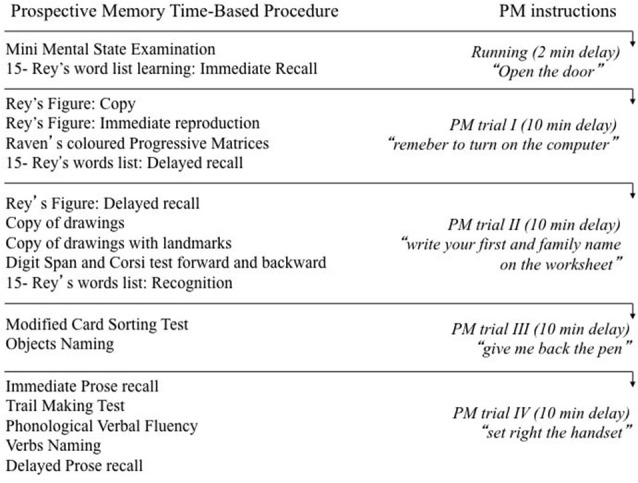
**The Figure illustrates the order of administration of the neuropsychological screening tests during the administration of the prospective memory procedure**.

The procedure is a slightly revised version of a task that was previously used with individuals who had MCI but not PD (Costa et al., 2015b). It consists of five consecutive trials, which are administered in approximately 50 min during administration of the neuropsychological screening battery. First, a running trial is performed. At the beginning of this trial the examiner instructs the subjects (who are tested individually) to perform the action after 2 min have elapsed. They have to repeat the examiner’s instructions aloud. At the end of this trial the examiner discusses the difficulties the subjects have encountered in performing the task. After this trial, four other consecutive trials are run. At the beginning of each trial, the examiner instructs the subjects to perform an action after 10 min have elapsed. Only this 10-min trials were considered to evaluate performance. The subjects are informed that when the time expires they will have up to 2 min to initiate the action (a “forgiveness period”) and that they may then interrupt the ongoing task. This delay was the same for all subjects. During the delay interval the subjects are engaged in performing the neuropsychological tests of the screening battery (Figure 1). In particular, the sequence of events is programmed so as to avoid synchrony between the moment when the prospective intention has to be retrieved and the moment when the recall phase of the episodic memory tests of the neuropsychological battery have to be performed. If the subjects start to perform the required actions within this time limit, the examiner records the action. If the subjects do not engage in any action at the expiration of the 2-min forgiveness period, the examiner reminds them: “Do you remember that at this point you were supposed to do something?” If the response is affirmative, the examiner records the action carried out. The same procedure is repeated for all trials.

We computed one score for recalling the intention to perform the actions (prospective component) and one for correctly executing the actions (retrospective component). If the intention was recalled, we gave a score of 1 for each spontaneously activated intention and 0 if the intention was not recalled (score range 0–4). For action performance, regardless of whether or not the intention was spontaneously recalled or had to be cued, we assigned a score of 1 if the action was performed correctly, a score of 0.5 if performance was partially correct and a score of 0 if the action was completely incorrect or lacking (score range 0–4).

All PD patients were assessed approximately 60–90 min. after they had received their dose of dopamine replacement drug.

## Statistical Analysis

A general MANOVA and subsequent univariate ANOVAs were performed to compare the three experimental groups on the scores they obtained on the tests of the neuropsychological screening battery. In the case of significant main effects *post hoc* Tukey HSD were performed.

To compare PM performance between PD patients with MCI, PD patients without MCI and HC subjects, a mixed ANOVA was carried out with Group (PD patients with MCI vs. NPD vs. HC) as between factor and Task Component (prospective vs. retrospective) as within factor. In the case of significant main effects, Tukey HSD *post hoc* tests were performed. To investigate whether PD patients’ PM performance predicts Pill questionnaire score, taking into account the weight of other relevant factors, the forward linear regression model was applied. The Pill score was entered in the model as dependent variable and the prospective and retrospective component scores of the PM task were entered as independent variables. According to the rule of thumb, to maintain a ratio of 10 subjects for each independent variable included in the regression model, with a sample of 81 subjects a maximum of 8 independent variables should be included. Therefore, we reduced the number of the other possible covariates by selecting one measure for each domain. Specifically, we included the UPDRS part-III to assess severity of extrapiramidal symptoms, the AES for apathy evaluation, the BDI as depression measure, the categories achieved on the MCST as measures of executive functioning, the delayed Prose Recall score as episodic memory measure and the MMSE score as an index of global cognitive functioning. The regression analysis was executed in a first step on the whole PD group and, in a second step, only with PD patients who had MCI.

A mixed ANOVA was also performed to compare PM scores between PD patients with single domain MCI who exhibit a dysexecutive vs. amnesic cognitive profile.

Pearson’s correlation analyses were performed to examine the relationship between PM scores and scores on the tests of the neuropsychological screening battery that investigate episodic memory and executive functions.

## Results

### Between Group Comparisons on the Tests of the Neuropsychological Screening Battery

Results of the general MANOVA showed a significant effect (Roy’s *root*_(21,79)_ = 6.68; *p* < 0.001).

Results of univariate comparisons are reported in detail in Table 2. To summarize these results, a significant between groups effect with *p* < 0.05 was found for all tests included in the analysis, with the exception of the Digit Span Forward and Backward, the Corsi Block Tapping test Forward and Prose Memory Immediate Recall. *Post hoc* Tukey HSD comparisons showed that PD patients with MCI had significant lower scores on HC on all tests (all *p* consistently <0.05) with the exception of Prose Memory Delayed Recall (*p* = 0.06) and verbs (*p* = 0.09) naming and Corsi Block Tapping test Backward (*p* = 0.18). Moreover, PD patients with MCI had significant poorer performance than PDN on all tests (all *p* consistently <0.05). *Post hoc* comparisons between HC and PDN groups did not reveal any significant effect (*p* range: 1–0.09).

**Table 2:** **Average scores obtained by subjects of the three experimental groups with results of univariate ANOVAs are reported**.

Neuropsychological screening battery	PD patients with MCI	PD patients with MCI	Healthy controls controls	*p* values
	Mean (SD)			
*Episodic Memory*
15-Rey’s Word List—Immediate Recall	29.8 (9.1)	41.4 (7.3)	46.0 (9.0)	<0.001
15-Rey’s Word List—Delayed Recall	5.8 (2.4)	9.0 (2.2)	9.7 (2.3)	<0.001
Rey’s Figure—Immediate Recall	10.0 (6.3)	16.7 (6.8)	17.0 (7.1)	<0.001
Rey’s Figure—Delayed Recall	9.2 (5.9)	16.4 (9.2)	16.4 (6.1)	<0.001
Prose Memory—Immediate Recall	5.1 (1.5)	5.5 (1.4)	5.9 (1.2)	0.097
Prose Memory—Delayed Recall	4.8 (1.6)	5.6 (1.4)	5.7 (1.1)	0.016
*Attention and Short-term Memory*
Trail Making Test—Part A	66.5 (27.6)	46.4 (14.8)	37.2 (13.9)	<0.001
Digit Span Forward	5.5 (1.1)	5.9 (1.0)	5.9 (0.8)	0.11
Corsi Block Tapping test Forward	4.7 (0.9)	5.1 (0.8)	4.9 (0.8)	0.13
Digit Span Backward	3.8 (1.5)	4.3 (0.9)	4.2 (0.7)	0.12
Corsi Block Tapping test Backward	4.1 (1.0)	4.7 (0.8)	4.6 (0.7)	0.022
*Executive Functions*
Modified Card Sorting Test—Categories Achieved	3.9 (1.9)	5.7 (0.5)	5.7 (0.7)	<0.001
Modified Card Sorting Test—Perseverative Errors	5.2 (5.2)	2.1 (2.0)	2.0 (2.3)	0.001
Trail Making Test—Part B	227.8 (105.3)	131.2 (46.9)	102.1 (36.6)	<0.001
Raven’s Colored Progressive Matrices	25.6 (4.5)	30.4 (3.9)	30.4 (4.5)	<0.001
Phonological Verbal Fluency	25.6 (9.9)	35.6 (9.5)	35.7 (5.4)	<0.001
*Language*
Objects Naming	28.6 (3.1)	29.8 (0.5)	29.8 (0.6)	0.019
Verbs Naming	26.1 (3.2)	27.5 (0.9)	27.4 (0.8)	0.013
*Visual-spatial Functions*
Rey’s Figure—Copy	27.2 (5.5)	30.4 (4.3)	32.4 (2.6)	<0.001
Copy of Drawing	8.6 (1.5)	9.8 (1.3)	10.4 (1.3)	<0.001
Copy of Drawing with Landmarks	66.9 (3.7)	68.7 (2.1)	68.9 (1.7)	0.009

### Between Group Comparisons of PM Performance

**Figure 2:**
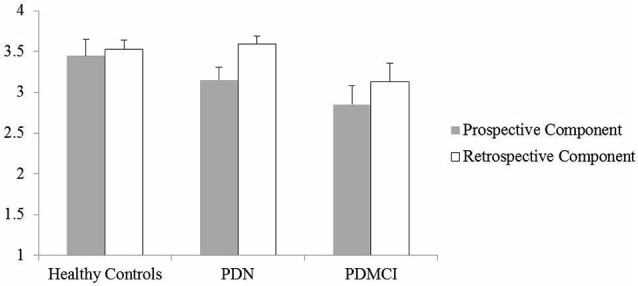
**The Figure illustrates the average performance accuracy of the individuals of the three experimental groups on the prospective and retrospective component of the prospective memory (PM) procedure**. Vertical bars represent standard errors.

Figure 2 illustrates performance of the four experimental groups on the PM procedure.

Results of the mixed ANOVA show a main effect of the Group (*F*_(2,98)_ = 4.32; *p* = 0.016) and Task Component (*F*_(1,98)_ = 5.99; *p* = 0.016) factors. By contrast, the interaction between the two main factors did not reach statistical significance (*F*_(1,98)_ = 0.79; *p* = 0.45). The main effect of the Task Component documents that in the whole sample the prospective component score was significantly lower than the retrospective component score (mean = 3.07; SD = 0.99 and mean = 3.36; SD = 0.82, respectively). Tukey HSD tests made to qualify the Group effect documented that, independently from the PM task component considered, PD patients with MCI (prospective component: mean = 2.85; SD = 1.05; retrospective component: mean = 3.14; SD = 1.01) performed worse than HC (prospective component: mean = 3.45; SD = 0.89; retrospective component: mean = 3.53; SD = 0.52; *p* = 0.034; Cohen’s *d* = 0.57) and tended to perform worse than PDN individuals (prospective component: mean = 3.15; SD = 0.91; retrospective component: mean = 3.59; SD = 0.55; *p* = 0.064; Cohen’s *d* = 0.43). The comparison between PDN and HC groups showed, instead, that the result was quite far from the statistical significance (*p* = 0.84; Cohen’s *d* = 0.17).

### Factors Predicting the Pill Questionnaire Score

#### Regressions Performed on the Whole PD Group

In the first step of this analysis the MCST-categories score entered the regression equation (*F*_(1,80)_ = 19.7; *p* < 0.001; *R*^2^ = 0.20) with a positive correlation with the dependent variable (Beta = 0.45; Pearson’s *r* = 0.45; *t* = 4.44; *p* < 0.001). This shows that better performance on this test is associated with better self-management of pharmacological therapy. In the second step the prospective component score of the PM procedure significantly contributed to the model (*R*^2^ change = 0.074; *F*_(2,80)_ = 14.8; *p* < 0.001), also in this case it correlated positively with the criterion (Beta = 0.27; Pearson’s *r* = 0.30; *t* = 2.83; *p* = 0.006), thus documenting that better intention retrieval is associated with better scores on the Pill questionnaire. The last variable entering the regression equation was, in the third step, the AES score (*R*^2^ change = 0.041; *F*_(3,80)_ = 11.91; *p* < 0.001); in this case it correlated inversely with the dependent variable (Beta = −0.21; Pearson’s *r* = −0.27; *t* = −2.19; *p* = 0.032). This documents that lower apathy rates are associated with better Pill questionnaire scores. None of the other factors tested contributed significantly to the model (partial correlations: retrospective component of the PM task = −0.05; UPDRS part-III = −0.05; BDI = 0.11; delayed Prose Recall = −0.01; MMSE = 0.05).

#### Regressions Performed Selectively on the MCI Sample

**Figure 3:**
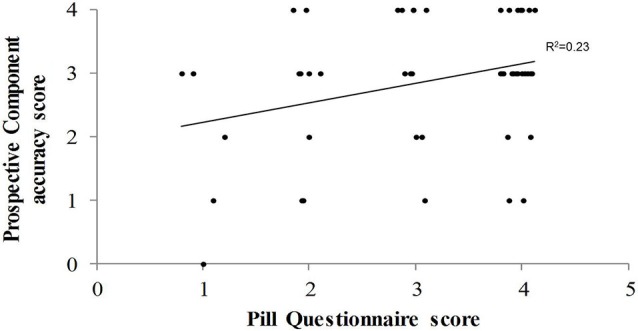
**The Figure illustrates, in the PD patients with MCI, the correlation between the score on the Pill questionnaire and accuracy score on the prospective component of the prospective memory procedure**.

These regressions were executed to verify the above findings in the MCI group. The dependent variable was the same as above (i.e., score on the Pill questionnaire). In this case, however, the independent variables were those for which a significant effect had been found in the previous analysis, that is, the categories achieved on the MCST, the AES score and accuracy on the prospective component of the PM task. Results substantially confirmed the findings on the whole PD sample, with the exception of the AES, which no longer approached statistical significance (Beta = −0.21; *t* = −1.61; *p* = 0.12). In particular, in the first step the MCST-categories score (*F*_(1,46)_ = 6.87; *p* = 0.012; *R*^2^ = 0.13), and in the second step the prospective component score of the PM task (*F*_(1,45)_ = 6.89; *p* = 0.002; *R*^2^ = 0.23; Figure 3) significantly entered the regression equation. Also in these cases the two explicative factors correlated positively with the dependent variable (with Beta = 0.36; and Beta = 0.32, respectively).

### Relationship Between PM and Executive Functioning in the MCI Sample

#### Comparisons Between PD Patients with Executive and Amnestic Cognitive Profile within the Single Domain MCI Group

Results of the mixed ANOVA do not show any significant effect (Group: *F*_(1,18)_ = 0.07; *p* = 0.79; Task Component: *F*_(1,18)_ = 2.97; *p* = 0.10; Group*Task Component interaction (*F*_(1,18)_ = 0.97).

#### Correlation Between Scores on Tests of the Neuropsychological Screening Battery that Investigate Episodic Memory and Executive Functions and PM Scores

The following tests were considered for episodic memory: Delayed Recall of a 15-Word List and Prose Memory Delayed Recall. The following tests were taken into account for the executive domain: Phonological Word Fluency, MCST (both categories achieved and perseverative errors) and the Trail Making Test (Part B—Part A). Correlation analyses were performed separately for the prospective and retrospective component of the PM task. In order to avoid the risk of alpha inflation, Bonferroni’s correction was applied to these analyses. Accordingly, with six correlations the *p* value for considering a correlation as significant was set at 0.008 (i.e., *p* = 0.05/6).

Results of the correlation analyses did not reveal any significant effect both for the prospective component (*r* ranged from −0.09 to 0.12, and *p* ranged from 0.83 to 0.40) and the retrospective component (*r* ranged from 0.06 to 0.34 and *p* ranged from 0.81 to 0.018).

## Discussion

This study was aimed at investigating time-based PM functioning and its relationship with autonomous management of medication in PD patients with and without MCI. The first main finding of the study is that PD patients with MCI performed significantly worse than HC on both the prospective and retrospective components of a time-based PM procedure. Moreover, with respect to the PDN group (i.e., PD patients who showed no significant cognitive impairment on a standard neuropsychological examination), the MCI group tended to perform worse on the PM task. By contrast, no significant PM performance difference was found between PDN and HC groups. It is unlikely that the above differences between the PD sub-groups were due to the clinical characteristics of the samples, as patients in the various PD subgroups were comparable for duration and severity of the disease and dopamine dosage.

To the best of our knowledge, this is the first study that has investigated time-based PM abilities in PD patients with MCI. Therefore, the results add new valuable information to the extant literature because they document that in PD patients time-based PM weakness is related to the presence of MCI. Indeed, previous studies examined time-based PM functioning in PD patients and demonstrated that their performance was significantly poorer than that of healthy controls (Costa et al., 2008b; Raskin et al., 2011; but see Katai et al., 2003 for partially divergent results). In those studies, however, the possible effect of MCI was not investigated. Very interestingly, our findings are substantially in line with the results of a previous study in which event-based PM performance was found to be significantly decreased in PD patients with MCI compared to PD patients without cognitive impairments who, in turn, performed as well as healthy controls (Costa et al., 2015a). Taken together the current and previous findings provide convergent evidence of a strict association between PM deficits and MCI in PD patients, thus investigating PM functioning might help significantly in discriminating between MCI and non-MCI in PD. From a clinical perspective, this is a very important observation. In fact, the presence of MCI is associated with increased risk of dementia in the affected individual (Janvin et al., 2006; Litvan et al., 2012). Therefore, the early identification of MCI in PD should be a main goal to improve management and treatment of the disease (Emre et al., 2014).

However, it should be noted that we did not find a clear dissociation between the prospective and retrospective component of the PM task. Indeed, PD patients with MCI appear to be equally impaired in both PM components. Previous studies showed that PD patients, compared with both healthy controls and PD patients without MCI, have reduced performance on traditional episodic memory tests (Emre et al., 2007). Two main hypotheses have been advanced to account for such an evidence. The first hypothesis sees memory disorders in these patients as a consequence of consolidation failure. This hypothesis is grounded on findings showing that individuals with PD have lower performance than healthy controls in recognition and semantic cued recall tasks (Higginson et al., 2005; Davidson et al., 2006; Cohn et al., 2010). The second hypothesis views memory weakness as a consequence of encoding or retrieval failures. Data evidencing that PD patients significantly benefit by a semantic cueing of studied information and that their performance significantly improves in recognition tests sustain this hypothesis (Dujardin and Laurent, 2003; Emre et al., 2007). The pattern of memory disorders in PD patients with MCI is, indeed, poorly investigated. In a previous study we documented that PD patients with MCI, compared to non PD amnestic MCI individuals, significantly improved their memory recall in the Free and Cued Selective Reminding test after the semantic cue presentation (Costa et al., 2014). This finding could support the hypothesis that, in these individuals, memory disorders are mainly related to retrieval failure, likely as a consequence of reduced efficiency of frontal-striatal networks (Cools, 2006). However, in the current study, we did not find a clear relationship between PM performance and scores on the executive tests (and episodic memory tests) of the neuropsychological battery. Moreover, we did not find a clear difference on the PM performance pattern between PD patients with disexecutive and amnestic neuropsychological profile. This null finding could be due to the relatively low sample size of the single-domain MCI groups (*n* = 11 and *n* = 9, respectively). This issue is undoubtedly worth of further investigation in larger samples of PD patients with MCI.

The second main finding of this study also shows that PM assessment can provide some relevant information about patients’ functional autonomy. We applied a linear regression model to the data to investigate whether PM performance predicts PD patients’ ability to manage their own medication; we also took into account the potential effect of other cognitive, affective and motor factors. We entered the Pill questionnaire score as the dependent variable. The Task Force of the Movement Disorder Society (Dubois et al., 2007b) recommends that this tool be used to assess the impact of cognitive impairments on functional abilities in the PD population. It is held that the Pill questionnaire overcomes the limits of other instruments (e.g., ADL and IADL), which can be affected by PD-related motor symptoms (Dubois et al., 2007b). We found that the best model for predicting the Pill questionnaire score included accuracy on the prospective component of the PM task, number of categories achieved on the MCST and AES score. When the PDN group was excluded from the analysis (and only the MCI sample remained), the PM prospective component and the MCST scores remained significant predictors and the AES score effect was no longer significant. The relationship between the explicative variables and the criterion was in the expected direction, that is, better scores on the former were associated with better scores on the latter.

Our finding of a significant association between PM performance and PD patients’ autonomous management of their own medication was expected. Indeed, taking prescribed drugs at the correct time depends on the ability to monitor the time course and to spontaneously retrieve the related intention by interrupting the ongoing activity. This condition is quite similar to that represented by our PM procedure. In this regard, it is noteworthy that accuracy on the prospective (but not on the retrospective) component predicted the Pill questionnaire score. In fact, the prospective component of our procedure was completely self-guided as it specifically required that subjects autonomously shift their attention from the ongoing task to performing the planned action in the right time window without any explicit prompting by the examiner. Instead, the retrospective component required less self-management of the task. In fact, if the subject did not autonomously begin to perform the action (i.e., prospective component score = 0), he was reminded by the examiner that he was expected to perform some actions at the expiration of the 10-min period.

Our result was also expected on the basis of previous data suggesting that in PD patients there is a relationship between PM functioning and functional abilities (Pirogovsky et al., 2012; Costa et al., 2015a). However, with respect to previous findings the current result clarifies that this association is significant in PD patients with MCI independently from the effect of other cognitive (i.e., executive and episodic memory), affective (depression and apathy) and motor symptoms. In this regard, the finding that PM accuracy entered the regression model together with the MCST score, significantly improving the amount of variance explained by the model itself, is of particular interest. Indeed, it is generally held that the prospective component of a PM task, such as the one used here, depends strictly on the correct functioning of the executive system (Costa et al., 2011a; McDaniel and Einstein, 2011). Strategic monitoring of the time passing and shifting from the ongoing activity to intention retrieval are particularly stressed. Coherently, data obtained from healthy subjects (Burgess et al., 2011; Costa et al., 2011b, 2013b; Cona et al., 2015) and neurological patients (Kinsella et al., 1996; Fish et al., 2010; Carlesimo et al., 2014) document that the ability to spontaneously retrieve the intention during a PM task is associated with prefrontal cortex activity. However, as discussed above, the PM procedure resembles a multitasking condition in that it requires that subjects keep in memory and autonomously retrieve the intention to perform an action while performing an intercurrent ongoing activity. This condition seems substantially different from that proposed in the MCST and other classical executive tests and is probably more akin to daily living functioning. As a matter of fact we did not find significant correlations between scores on the executive tests of the neuropsychological screening battery and PM scores. Based on this observation, we can conclude that the additional amount of variance explained by the regression model by entering the PM score is related to this own characteristic of the PM procedure. In agreement with results of previous research (Pirogovsky et al., 2012; Costa et al., 2015a), this conclusion supports the ecological validity of PM tasks.

In conclusion, the results of this study demonstrate that in PD patients time-based PM weakness is specifically related to the presence of MCI. This finding indicates the importance of PM assessment to identify MCI during the course of PD. In this regard, it should be noted that in the current study we administered a PM task that was incorporated in the standard neuropsychological battery. This means that this task has the great advantage of not requiring significant additional time to assess PM. The same data indicate that the decreased efficiency of the time-based PM processes is associated with reduced independent behavior. Indeed, in PD patients dopamine therapy is often very complex and requires that doses be taken at different times of the day. Missing or repeatedly delaying medication may lead to a significant worsening of the patient’s condition. This observation further strengthens the potential importance of planning training programs aimed at improving PM abilities in PD patients with MCI.

## Author Contributions

AC, GAC, SZ, AP and CC made substantial contributions to conceptualizing the work and to acquiring, analyzing and interpreting the data; they drafted the work or revised it critically for important intellectual content; they gave their final approval of the version to be published; and they also agreed to be accountable for all aspects of the work and to ensure that questions related to the accuracy or integrity of any part of the work are appropriately investigated and resolved. FS made a substantial contribution to data acquisition; drafted the work or revised it critically for important intellectual content; gave final approval of the version to be published; and agreed to be accountable for all aspects of the work by ensuring that questions related to the accuracy or integrity of any part of the work are appropriately investigated and resolved.

## Conflict of Interest Statement

The authors declare that the research was conducted in the absence of any commercial or financial relationships that could be construed as a potential conflict of interest.
